# Rational design of an essential diagnostics network to support Universal Health Coverage: a modeling analysis

**DOI:** 10.1186/s12913-022-08558-2

**Published:** 2022-10-01

**Authors:** Lee F. Schroeder, Yvonne Dei-Adomakoh, Kristen DeStigter, Emmanuel O. Idigbe, John Flanigan, Priscilla Mawuli Awo Ekpale, Ernest Adjei, Lina Roa, Michael L. Wilson, Susan Horton

**Affiliations:** 1 Department of Pathology & Clinical Labs, University of Michigan Medical School, NCRC, Bldg. 35, 2800 Plymouth Road, Ann Arbor, MI 48109-2800 USA; 2 Department of Haematology, University of Ghana Medical School, Accra, Ghana; 3 University of Vermont Larner College of Medicine, Vermont, USA; 4 Human Development and Public Health Initiative, Lagos, Nigeria; 5 National Cancer Institute, Bethesda, MD USA; 6 Department of Pharmacy, Korle Bu Teaching Hospital, Accra, Ghana; 7 Department of Pathology, Komfo Anokye Teaching Hospital, Kumasi, Ghana; 8 Department of Obstetrics and Gynecology, University of Alberta, Edmonton, Canada; 9 Denver Health and Hospital Authority, Denver, CO USA; 10 School of Public Health Sciences, University of Waterloo, Waterloo, Canada

**Keywords:** Diagnostics, Universal Health Coverage, Laboratories, Imaging, Essential Diagnostics List

## Abstract

**Background:**

Diagnostic investigations, including pathology and laboratory medicine (PALM) and radiology, have been largely absent from international strategies such as the Sustainable Development Goals. Further, there is little international guidance on which health system tiers different diagnostics should be placed, a critical step in developing a country-level diagnostics network. We describe a modeling strategy to produce tier-specific diagnostic recommendations based on disease burden, current treatment pathways, and existing infrastructure in a country.

**Methods:**

The relational model assumes that diagnostics should be available at the lowest tier where patients might receive medical management. Using Ghana as an exemplar, the 20 diseases forecasted by 2030 and 2040 to cause the greatest burden in low- and middle-income countries were mapped to three generalized tiers in the Ghanaian health system (Primary, Secondary, and Tertiary care) for three levels of each disease (triage, uncomplicated, and complicated). The lowest tier at which a diagnostic could potentially be placed was restricted by existing infrastructure, though placement still required there be a medical justification for the diagnostic at that tier.

**Results:**

The model recommended 111 unique diagnostic investigations with 17 at Primary tier, an additional 45 at Secondary tier and a further 49 at Tertiary tier. Estimated capital costs were $8,330 at Primary tier and between $571,000 to $777,000 at Secondary tier. Twenty-eight different laboratory tests were recommended as send-outs from Primary to Secondary tier, and twelve as send-outs to Tertiary tier.

**Conclusions:**

This model provides a transparent framework within which countries can customize diagnostic planning to local disease priorities, health system patient treatment pathways, and infrastructural limitations to best support Universal Health Coverage.

**Supplementary Information:**

The online version contains supplementary material available at 10.1186/s12913-022-08558-2.

## Background

For decades, diagnostics have remained relatively invisible in global health policy as compared to medicines and vaccines. They are not mentioned in important international initiatives such as the Sustainable Development Goals [1, 2], and this has potentially contributed to poor capacity in many settings [3, 4]. The World Health Organization issued the first Essential Medicines List (EML) in 1977, and has recently published the first Essential Diagnostics List (EDL) in 2018 [5]. The resource could play an important role in improving the delivery of diagnostic services. The WHO describes the EDL as an “evidence-based reference point for countries to develop their own national lists to guide how they choose and use IVDs. The EDL recognizes that IVDs are essential for advancing universal health coverage (UHC), addressing health emergencies and promoting healthier populations, which are the three strategic priorities of WHO’s thirteenth general programme of work covering 2019–2023” [6]. The EDL was developed, and is periodically updated, with support of the WHO Strategic Advisory Group of Experts on In Vitro Diagnostics (SAGE IVD). However, it should be noted that the EDL does not currently include diagnostic imaging (radiology).

Arguably, one of the consequences of developing the EML has been to prioritize medicines in an evidence-based way [7, 8], guide procurement, and nurture discussion of international trade agreements [7, 9]. More than 155 countries have created national EMLs (NEMLs) [8, 10], comprising the large majority of low- and middle-income countries and a few high-income countries. Currently, only India and Nigeria have published national EDLs (NEDLs) [11, 12]. India was the first country [12], and went beyond the WHO EDL by including imaging in addition to in-vitro diagnostics, stratification of diagnostics by different health system tiers, and identifying diagnostics that should be provided as “send-out” tests to higher-tier laboratories. Developing the Indian NEDL involved extensive stakeholder consultation and incorporation of the latest evidence on disease burden. A transparent framework for establishing and updating national EDLs (NEDLs) would be an important contribution to their wider adoption by countries. While the WHO EDL does distinguish tests that either do or do not require laboratories, more granular tier-specific recommendations are needed.

There have been previous efforts to outline tier-specific laboratory test [13–15] and imaging [16] allocation, relying on experience, expert opinion, and international guidelines. This manuscript describes a modeling approach to derive the first evidence-based allocation of essential diagnostics by health system tier including both laboratory tests and imaging. In order to address the gap in international guidance on tier-specific diagnostic recommendations, the model described here incorporates tier-specificity as a fundamental feature. It makes the assumption that diagnostics should be available at the lowest tier at which patients might receive medical management dependent on the results of the diagnostic. That strategy facilitates a critical mass of medical expertise and resources and reduces treatment abandonment while helping to lower out-of-pocket payments resulting from travel away from home. If a diagnostic investigation cannot be performed at the desired tier due to infrastructural limitations, the model assumes the use of “send-out” testing for laboratory diagnostics, provided results can be returned within a reasonable amount of time. This is an option used in the Indian NEDL and is the basis of an integrated laboratory network as described in the Maputo Declaration of 2008 by the World Health Organization Regional Committee for Africa [17].

An advantage of our modeling approach is that it permits a transparent and explicit integration of disease priorities in a country, current tier-specific patient treatment pathways, and existing infrastructure into a decision support framework for planning diagnostic networks. This work represents the full description of an expanded modeling effort that was presented in an earlier version as a brief outline in the Lancet Commission on Diagnostics in 2021 [4].

## Methods

The relational model developed here identifies tier-specific diagnostics to prioritize in health facilities of a national health system, focusing on Ghana as an exemplar. The tiers include Primary (e.g., health centers), Secondary (district-level hospitals), and Tertiary (regional and referral hospitals) levels. Diagnostics used for condition-related indications (e.g. diagnosis, monitoring for treatment response and identifying co-morbidities) are included as well as medication-related indications (e.g., monitoring toxicity of medicines and adjusting dosage). The disease priorities, termed here the GBD-20 are drawn from the top 20 diseases based on the Global Burden of Disease (GBD) projections for mortality and for years of life lost for 2030 and 2040 in LMICs as defined in 2018 (Table 1) [4, 18, 19]. The GBD-20 was chosen (instead of the GBD projections for Ghana specifically) so that this model would in general hold some relevance to a wider set of countries. Of course, individual countries can add other priority conditions based on local epidemiology.

**Table 1:** Top 20 conditions responsible for years of life lost, GBD 2030 and 2040

Condition	GBD term (if different)	Appears in GBD lists
Acute coronary syndrome	Ischemic heart disease	Global 2030, 2040; LMIC 2030, 2040
Antenatal care	Neonatal preterm birth, Neonatal encephalopathy^b^	Global 2030, 2040; LMIC 2030, 2040
Breast cancer		Global 2040
Cerebrovascular disease		Global 2030, 2040; LMIC 2030, 2040
Chronic kidney disease		Global 2030, 2040; LMIC 2030, 2040
Colorectal cancer		Global 2030, 2040; LMIC 2040
COPD		Global 2030, 2040; LMIC 2030, 2040
Dementia	Alzheimer’s disease (and related dementias)	Global 2030, 2040; LMIC 2030, 2040
Diabetes		Global 2030, 2040; LMIC 2030, 2040
Diarrhea acute invasive bacterial	Diarrheal diseases	Global 2030, 2040; LMIC 2030, 2040
HIV		Global 2030, 2040; LMIC 2030, 2040
Hypertensive heart disease		Global 2030, 2040; LMIC 2030, 2040
Liver Cancer		Global 2030, 2040; LMIC 2030, 2040
Lung cancer	Lung cancer; also tracheal, bronchus & lung cancer	Global 2030, 2040; LMIC 2030, 2040
Malaria		Global 2030; LMIC 2030, 2040
Pneumonia, severe	Lower respiratory infection	Global 2030, 2040; LMIC 2030, 2040
Preeclampsia, HELLP^a^	Condition screened for during antenatal care	Global 2030; LMIC 2030, 2040
Trauma	Falls	Global 2040
	Interpersonal violence	LMIC 2030
	Road injuries	Global 2030, 2040; LMIC 2030, 2040
	Self-harm	Global 2030, 2040; LMIC 2040
Tuberculosis		Global 2030, 2040; LMIC 2030, 2040

^a^ Hemolysis, elevated liver enzymes and low platelets: a late-pregnancy syndrome

^b^ Neonatal preterm birth and neonatal encephalopathy (in GBD-20) can be prevented in part through antenatal care but are not otherwise addressed in the analysis; congenital defects (also in GBD-20) not included in analysis

Much of this model is founded upon a relational model that associates diagnostics to conditions and medicines from the World Health Organization Essential Medicines List, derived from expert databases [20]. The focus of that model was to identify diagnostics indicated for either prescribing the medicines in the WHO EML or for managing the diseases addressed by medicines in the EML. For the present study, the model was expanded to include diagnostic imaging (radiology) using published guidelines [16] as well as to produce health system tier-specific recommendations. Also, instead of beginning with the EML, the model begins with the GBD-20. Antenatal care was added to address two conditions on the top 20 GBD list through healthy pregnancy outcomes [21]. The term diagnostic investigations will be used to refer to both diagnostic tests (the term used in PALM) and examinations (the term used in radiology).

**Fig. 1:**
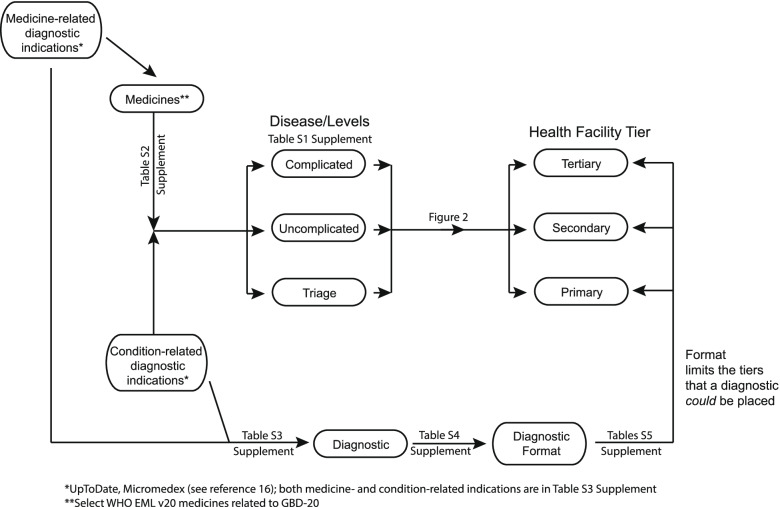
Summary of the relational model to recommend tier-specific diagnostics within a health system

The model relies on several sets of assumptions and relationships as summarized in Fig. 1. First, the management of conditions is categorized into three levels, namely triage (T), management of the uncomplicated condition (U), and management of the condition with complications (C), as defined in Supplementary Table S1. Triage refers to identification of those individuals who potentially have the condition in order to refer them, often to a higher-level tier, for diagnosis and management. Different health systems may choose to address a particular level of a particular condition (e.g., management of uncomplicated diabetes mellitus) at different health facility tiers.

For the purposes of this analysis, Ghana was used as an exemplar, though the results should be relevant to many countries. Ghana was chosen because three of the co-authors are Ghanaian working in Ghana, and another three have done work in the country. Further, a number of studies have been published to landscape diagnostic capacity in the country, providing some context [22–25] and the Ghanaian National Health Insurance Scheme [26] reimburses a set of diagnostic investigations that could be informed by this study.

**Fig. 2:**
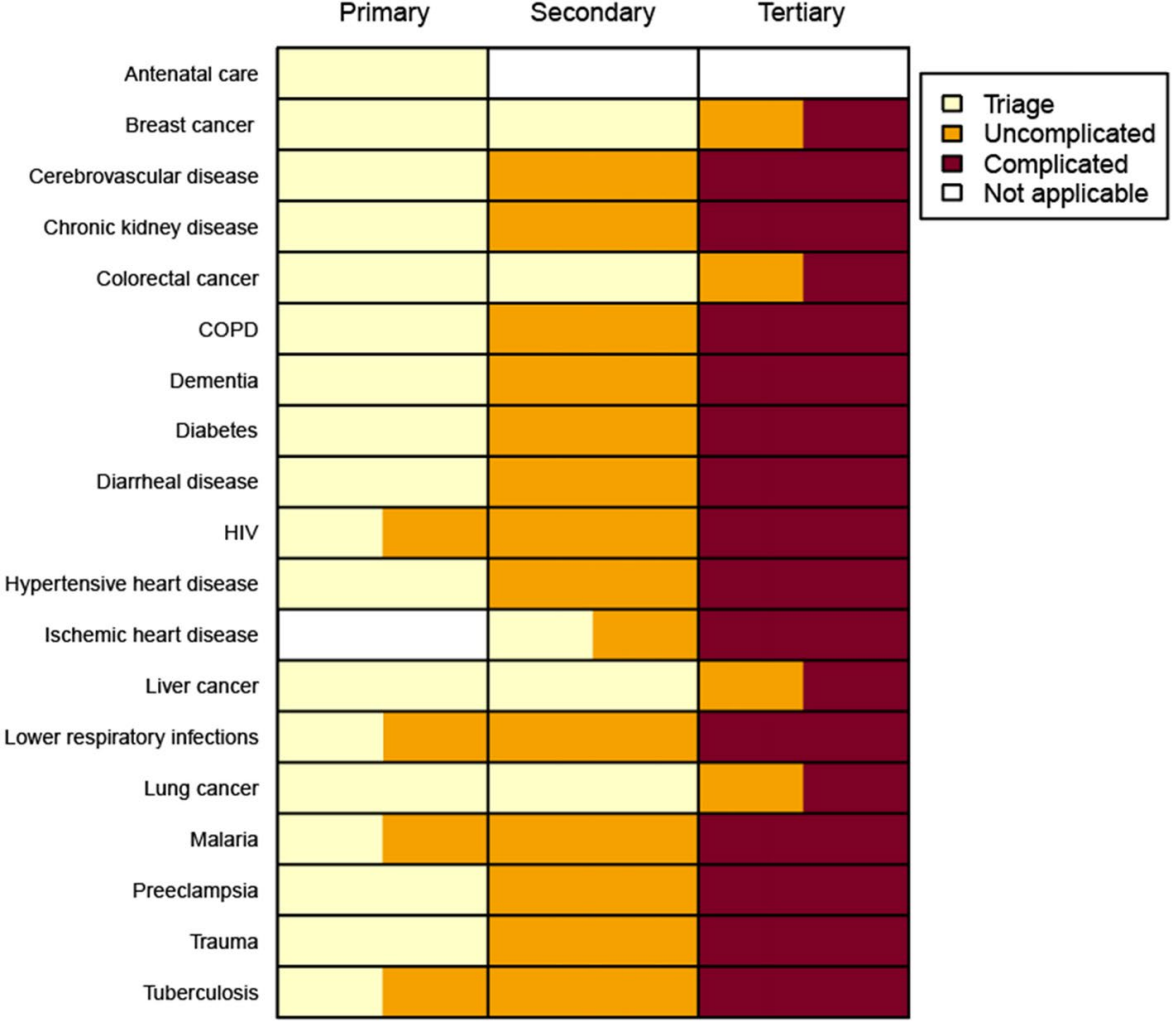
Map of GBD conditions incorporated into the model, with assignment of condition level (Triage, Uncomplicated, Complicated) with health facility tier (Primary, Secondary, Tertiary)

The first step in model-building was to map the GBD-20 condition-levels to health facility tiers according to current treatment practices in Ghana (Fig. 2). A future goal is to build a tool to allow users to move treatment of specific conditions between tiers and explore the associated change in the diagnostic template.

Second, each condition (and level, in this case uncomplicated or complicated) was assigned to relevant medicines from the WHO EML v20 [27], with some medicines assigned to multiple conditions (Supplementary Table S2). Medicines from the WHO EML v20 that are currently in use in Ghana were selected. For example, amoxicillin was assigned to uncomplicated pneumonia, while fifth-generation cephalosporins were assigned to complicated pneumonia. If the WHO EML was not sufficiently specific, authors’ opinion was used to make specific assignments. As no medicines on the WHO EML were appropriate for hepatocellular carcinoma, sorafenib was added. Likewise, gefitinib (which is now on WHO EML v21 [28]) was added for EGFR positive non-small cell lung cancer.

Third, diagnostics were assigned to specific conditions according to Schroeder et al., [20] and for the purposes of this model, also to a specific level of each condition (e.g., triage, uncomplicated, or complicated, using guidelines as far as possible, supplemented by authors’ expert opinion; Supplementary Table S3). If a diagnostic was required to in fact determine the level of the condition (uncomplicated vs complicated), the diagnostic was assigned to the uncomplicated level. Finally, for laboratory tests, each diagnostic was assigned to one or more formats (e.g. rapid diagnostic test, automated immunoassay analyzer; Supplementary Table S4), and in turn, these formats were assigned a minimum tier at which they could potentially be placed due to needs for specialized equipment or facilities or personnel (Supplementary Table S5). Importantly, these assignments did not ensure a diagnostic *would* be available at a given tier, only that it *could* be placed at a given tier if patient management at that tier required the diagnostic.

Pricing of equipment was derived from personal communications with co-author EI for laboratory diagnostics and with KD for radiological equipment. UNICEF target product profiles [29] were used for glucometers and haemoglobinometers, and the College of American Pathologists CAP TODAY Product Guide [30] (median prices for each instrument category) for chemistry, immunoassay, haematology, coagulation, and blood gas instruments.

Role of the funding source: Bill and Melinda Gates Foundation did not play a role in study design; data collection, analysis, or interpretation; or in the writing or submission of the report.

## Results

**Fig. 3:**
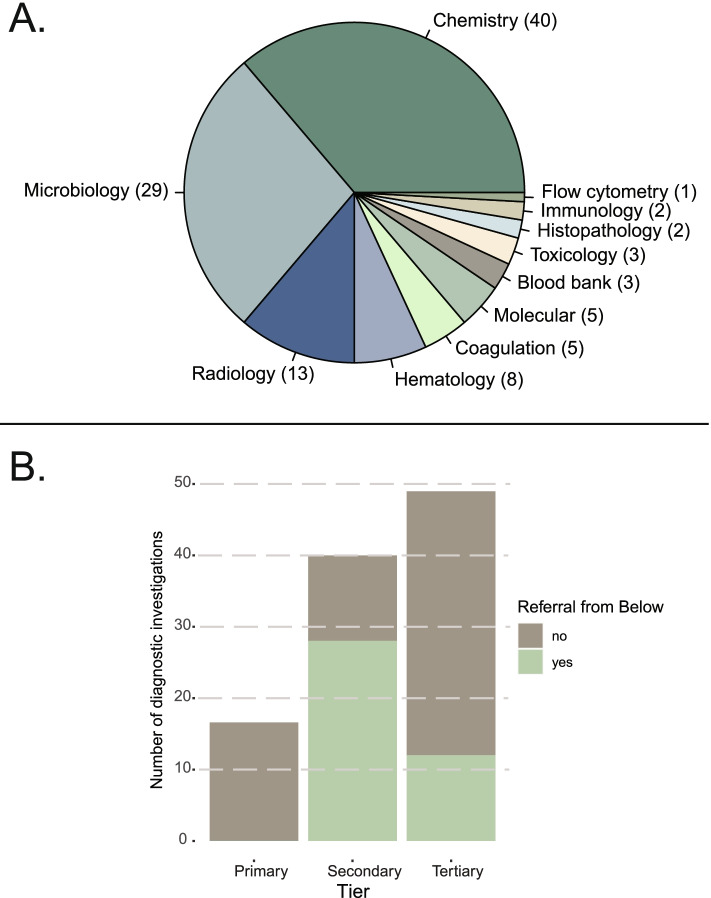
Diagnostics by category (**A**) and tier at which used (**B**). These categories do not take account of frequency of use, for example X-ray is used frequently for many different purposes, whereas some laboratory tests are used infrequently for a few specific purposes. Green bars in 3b represent laboratory tests that should be available at lower tiers but due to infrastructural limitations must rely on specimen transport from lower tiers to higher tiers

For the GBD-20, the model generated a list of diagnostics to be performed at each health facility tier and identified those that would ideally be placed at lower tiers but must rely on specimen transport (“send-out tests”), or referral of patients, due to logistical and infrastructural reasons (see Table 2 for a list of equipment needed to run the diagnostic investigations indicated; see Supplementary Table S6 for a complete list of diagnostic investigations). The model identified 111 unique diagnostic tests and examinations (not distinguishing different laboratory formats for the same analyte, e.g., HIV serology by rapid diagnostic test or automated immunoassay analyzer were counted as a single test), including 13 radiological examinations and 98 laboratory tests. The latter included tests in chemistry (40), microbiology (29), hematology (8), coagulation (5), molecular (non-microbiological) (5), transfusion testing (3), toxicology (3), anatomic pathology (2), immunology (2), and flow cytometry (1) (Fig. 3a).

**Table 2:** Model output describing instrumentation and equipment by tier

Primary tier	Secondary tier	Tertiary tier
Rapid diagnostic tests (RDTs) Glucometer Hemoglobinometer Basic microscopy: malaria, stool, urinalysis POC Ultrasound	RDTs Automated coagulation analyzer Automated chemistry analyzer Automated immunoassay analyzer Automated hematology analyzer ELISA Benchtop analyzers (e.g., blood gas, NAAT) Microscopy with stains (e.g., gram stains, AFB, trichrome, india ink) Bacterial culture (identification, antimicrobial susceptibility) Ultrasound X-ray	Additional analytes on automated analyzers Fungal & TB culture Automated nucleic acid analyzer HPLC Fluorescent spot (G6PD) Immunofluorescence microscopy Transfusion testing Flow cytometry Histopathology Immunohistopathology Cytopathology Advanced breast imaging Computed tomography Echocardiogram Fluoroscopy Basic interventional radiology Complex interventional radiology Mammography MRI Nuclear radiology Positron Emission Tomography

All diagnostics available at lower tier levels are also available at higher tier levels but are unlisted in this table. Tests may be performed in different formats at different tiers or even within the same tier, and placement of all formats may not be necessary

*Abbreviations: ELISA* Enzyme-linked immunosorbent assay, *G6PD* Glucose-6-phosphate-dehydrogenase deficiency, *AFB* Acid-fast bacteria, *NAAT* (nucleic acid amplification test), *POC* Point-of-care, *HPLC* High performance liquid chromatography

Of these same 111 diagnostic investigations, the model recommended 17 (including point-of-care ultrasound) at Primary tier, with an additional 45 (including ultrasound and X-ray) at Secondary tier, and a further 49 (including 10 advanced imaging capabilities) at Tertiary tier (Fig. 3b). Twenty-eight different laboratory tests were to be sent out from Primary to Secondary tier, and twelve were to be sent from lower tiers to Tertiary tier. Of the 111 diagnostic investigations, 20 were identified with medicine-related indications only (e.g., toxicity of medicine, or a companion diagnostic to a medicine).

When considering diagnostic need by condition in isolation of the other conditions, the condition with the most associated diagnostics was ischemic heart disease (36 diagnostics) and the least was dementia (7 diagnostics). Of course, many diagnostics are shared between conditions. When counting cumulative number of diagnostics, adding each condition sequentially (starting with the one requiring the most tests), there was a consistent increase in diagnostics per additional condition with a median of 4.5 diagnostics (Supplementary Figure S1).

Supplementary Table S7 presents estimates of the investments required in terms of equipment at each of the three levels, along with the minimum workforce skills. Although it would be desirable to also estimate the recurrent costs of operating diagnostic investigations at each level, that is not attempted here, since that depends critically on the volume of investigations for the target population, which depends in turn both on epidemiology but also on care-seeking behavior. The equipment costs for diagnostics at the Primary tier are modest ($2330 for laboratory; $6000 for portable ultrasound, excluding costs of service contracts and import duties). Equipment investments also require investments in training staff. The cost of setting up our recommended set of diagnostic capabilities at the Secondary tier, assuming one instrument of each type, is of the order of several hundred thousand dollars (anywhere from $296,000-$377,000 for the laboratory analyzers and $275,000-$400,000 for one ultrasound and an X-ray machine). This requires at minimum laboratory and imaging technicians (each with about 2 years of training), preferably some laboratory and imaging technologists (each with about 4 years of training), and supervision by (ideally, full-time on-site) a laboratory director (PhD or pathologist) and radiologist. Setting up diagnostic facilities at the Tertiary tier would involve significantly greater costs, with correspondingly greater needs for specialized staff, but these costs were not explicitly modeled. The investment costs modeled do not include the ongoing costs of staff salaries, reagents for laboratory tests, service contracts for laboratory and imaging equipment, and costs of maintaining quality (supervision, maintaining professional certification of staff and accreditation of equipment and procedures).

## Discussion

We have developed a relational model associating diseases, medicines, and diagnostics with the goal of creating tier-specific diagnostic placement recommendations for a country-level health system, using Ghana as an exemplar. The model, including radiology as well as pathology and laboratory medicine, recommended 111 unique diagnostic investigations with 17 at Primary tier, an additional 45 at Secondary tier and a further 49 at Tertiary tier. Estimated capital costs were $8,330 at Primary tier facilities and between $571,000 to $777,000 at the Secondary tier. Twenty-eight different laboratory tests were recommended as send-outs from Primary to Secondary tier, and twelve as send-outs to Tertiary tier.

The approach here is a forward-looking one, using the burden of disease projected for 2030 and 2040 for LMICs, which is very similar to the global projections. This was chosen as investments in diagnostics are likely to produce realizable gains only after years to decades. A finer disaggregation could be undertaken, for example for low-income countries where the epidemiological transition is least far advanced such that the burden from infectious diseases remains high and non-communicable diseases are less entrenched. The inputs to this model were based on practices and health system resources available in Ghana but due to our use of the GBD-20, the findings are likely generally applicable in other LMIC settings with similarly structured tiered health systems.

While the justification for diagnostics across tiers is multifactorial and due to patient management of multiple conditions according to Fig. 1, there are some themes that can be highlighted. Table 2 and Supplementary Table S6 indicate the basic diagnostic services that should be offered at each tier. At the Primary tier, diagnostics are driven in part by antenatal care, which requires (as part of WHO Recommendations for a Positive Pregnancy [21]) a variety of rapid diagnostic tests, dipstick tests and tests requiring basic equipment (hemoglobinometer, glucometer) [21]. The WHO Recommendations also include one ultrasound during the pregnancy which, in countries where this is feasible, could be undertaken at Primary tier using point-of-care ultrasound, provided staff had appropriate training. The tests recommended for pregnant women are also important for other patient populations to triage several conditions, both communicable and non-communicable. Also important in Ghana at the Primary tier, and many other countries in sub-Saharan African, is management of HIV, malaria, TB, and lower respiratory infections. Given the public health priority for these conditions, many point-of-care assays and microscopy not requiring substantial infrastructure have been developed and thus are able to be recommended at the Primary tier.

As depicted in Fig. 2, patients with uncomplicated levels of most conditions, with the exception of cancers, are assigned to be managed at the Secondary tier. The model shows the diagnostic services required to support Secondary tier patient management include basic automated hematology, chemistry and immunoassay analyzers, basic traditional bacteriology, X-ray, and ultrasound. Deployment of bacterial cultures, bacterial identification, and antimicrobial resistance testing requires significant training and resources, but is needed at the Secondary tier. Where infrastructure will not permit this, then a robust system of specimen transport is reasonable, given that bacterial cultures typically take a full day, or days, to develop. A number of diagnostics are also recommended at this tier to support clinical need of Primary tier patients, where infrastructure did not permit placement at lower tiers. This includes such testing as for lipids, pancreatic enzymes, liver function, renal function, hemoglobin A1c, blood typing (to support antenatal care) and X-ray examinations. These tests might also guide manufacturers when choosing new targets for developing point-of-care assays that could be employed in primary care facilities with limited laboratory resources.

Finally, Tertiary tier care is assumed to manage complicated versions of most conditions, as well as uncomplicated and complicated cancer management. At this tier, the model recommends advanced imaging capabilities (CT, PET, echocardiogram, advanced breast imaging, MRI, basic and complex interventional radiology, and nuclear radiology), companion diagnostics for personalized medicine in cancer, anatomic pathology including immunohistochemistry, blood transfusion testing, some basic therapeutic drug monitoring in addition to an expansion of tests from other services such as chemistry, immunology, microbiology and hematology. Many of the diagnostics at this level are identified as referral testing for patients at lower tiers, and again these might be considered by manufacturers when developing new point-of-care assays. In some cases, the instruments at this tier are of higher quality and can act as a reference when the point-of-care assays at the Primary tier are producing unexpected results, e.g., G6PD testing (which is indicated for identifying red blood cell enzyme deficiencies that could lead to hemolytic anemia when taking some medicines) or nucleic acid testing on automated analyzers as backup for testing in other formats at lower tiers.

Our model shows some similarities but also differences with the WHO EDL v3. Our model identifies a total of 16 tests plus ultrasound at the Primary tier. Our Primary tier recommendations are roughly equivalent to the EDL recommendations for facilities without laboratories [6]. That EDL subset includes 26 tests when counted using our diagnostic nomenclature (e.g., we count urinalysis dipstick as 1 diagnostic, but it is listed as 5 different diagnostics in the EDL). However, EDL v3 is not limited to the GBD-20 and many of the tests included on the list reflect that, such as tests for Chagas disease, cholera, hepatitis B, hepatitis C, influenza, sickle disease, strep pharyngitis, syphilis, and visceral leishmaniasis. On the other hand, some tests on our list for primary care are not on the EDL v3, including fecal occult blood testing or stool parasite microscopy. Some other tests in our Primary tier are in the EDL v3, but assigned to facilities with laboratories, e.g., fecal immunochemical testing, G6PD, and urine microscopy. Based on current assessment of infrastructure in Ghana, we concluded these tests could be offered at the Primary tier.

Another source of comparison is the WHO medical device technical series. There is one regarding cardiovascular disease and diabetes and another for cancer [31, 32]. While there is a third regarding reproductive, maternal, newborn and child health [33], many recommendations are similar to the WHO Recommendations for a Positive Pregnancy [21] that was used as input to our model. Using the nomenclature of our database, the WHO medical device technical series document for cardiovascular disease and diabetes lists 33 diagnostics (10 in the Primary tier, 17 additional in Secondary, and 5 additional in Tertiary). While we modeled more than just cardiovascular disease and diabetes, our final recommendations included all 33 of these diagnostics except triiodothyronine (T3) and thyroxine (T4). These were presumably included in the WHO document due to associations of thyroid dysfunction with dyslipidemia, alterations in cardiac output, and glycemic metabolism. These tests could reasonably be incorporated in our model by including a diagnostic indication for the co-morbidity of thyroid dysfunction with diabetes or cardiovascular disease. The WHO placed some diagnostics in the Primary tier that our model placed in Secondary: cardiac markers, B-type natriuretic peptide, coagulation (PT/PTT), d-dimer products. They also placed HbA1c and CBC in Primary, which we included as specimen transport from the Primary tier. Further, two diagnostics were placed in the Secondary tier by WHO but were placed in Tertiary in our model: CT scans and fluoroscopy. Each of these tier-differences are reflective of our assumption that patient triage and management for ischemic heart disease was placed entirely in Secondary tier and above and we did not include all cardiovascular disease (e.g., atrial fibrillation treated by warfarin was not considered in the model). The WHO medical device technical series also identifies medical devices for cancer [32] though the specific WHO diagnostic recommendations were not by tier but rather by ‘priority’, defined as requiring low, medium, or high resources. Therefore, no tier-specific comparison could be made. Nonetheless, all 18 diagnostics recommended in the document were included for placement in our model.

The national EDL model developed here should ideally link directly to UHC. If UHC covers specific population groups, for example with free access to treatment for children under the age of five, then diagnostics for this age-group should also be free and available in the public sector. If UHC covers specific medicines for some conditions, then the diagnostics associated with those conditions should also be covered by UHC. This benefits equity, otherwise affluent households who can afford to pay out-of-pocket for diagnostics will gain access to publicly funded treatment, whereas poor households will be excluded. Linking UHC coverage of diagnostics and treatment also ensures cost-effective allocation of public funding; without this, public funding may be wasted by inappropriate and unnecessary treatment. The benefits of linking provision of diagnostics, particularly point-of-care diagnostics, to provision of treatment have been seen for the major infectious diseases of global concern such as HIV-AIDS and malaria [34, 35].

Strengths of the approach here are that it builds on previous efforts relying more on expert opinion [13–15] by formally incorporating evidence on disease burden, evidence-based disease guidelines, and imaging diagnostics. The methodology also explicitly includes disease prioritization, which is not necessarily the case when the national EDL is generated from the national EML [20]. National EMLs are not always up to date, do not always rely on the most current evidence [36], and are not typically prioritized by burden of disease. The model used here also provides policy implications for workforce and equipment required at different tiers in the health system, which could inform costing studies. It can help plan the evolution of diagnostics networks, as the tier at which specific conditions can be managed evolves.

A limitation of the model as developed in this analysis is that it is based on only the GBD-20. However, as shown in Supplementary Figure S1, there is substantial overlap of testing needs between conditions and it is reasonable to think that there would be marginally decreasing diagnostic needs as additional conditions are added. Likewise, the model should be augmented with those diagnostics required for locally prioritized conditions. These can include infectious diseases (e.g., neglected tropical diseases), but also conditions depending on local variations in genetic background in the population (e.g., prevalence of sickle-cell anemia in regions such as West Africa) as well as conditions related to local food and health practices affecting disease (e.g., gastric cancers associated with local food preservation practices, and oral cancers associated with local chewing practices). Further, while our model is based on diagnostic recommendations in expert databases, they do not always include data on cost-effectiveness, a factor that will need to be considered at the country level. Likewise, accuracy of different formats for tests will vary, and this trade-off will need to be addressed during implementation.

Also, the model does not incorporate future changes in diagnostic technology that may alter which diagnostics can be feasibly used at particular levels in health systems. The greater future availability of point-of-care diagnostics, including self-sampling and self-testing, will change what is possible. The model also does not take account of population density, which may affect diagnostic placement. For laboratory testing that does not require a short turn-around time, it may be cost-effective to plan on specimen referral even when technology might permit placement at lower levels, particularly when testing volumes are expected to be low at lower tiers and loss to follow-up is less of a concern. Greater population density makes it more feasible (due to scale economies) to purchase larger analyzers to undertake a higher volume of tests, or to afford more expensive imaging equipment at lower-tier facilities. As a result, the Indian NEDL is likely to differ in some significant ways from those developed in sub-Saharan Africa, since greater population density and urbanization, and hence volume of patients at individual health facilities, can permit greater decentralization of management of conditions by health system tier. Finally, the model does not include functions other than management of conditions, for example it does not include surveillance for infectious diseases or other public health functions such as assuring the national blood supply for hospitals or environmental safety.

## Conclusions

Universal Health Coverage requires an integrated health system from primary through tertiary care. An integrated health system must include a tiered laboratory and radiological network, providing services along the care-continuum. The need for this has been described in numerous international declarations and guidelines. The model presented here provides guidance as to how to allocate diagnostics across different tiers in health systems and can be adapted to individual country situations. There is a growing field of diagnostic network optimization [37], designed to assist in cost-effective placement of diagnostics across a country. The model presented here may be useful in conjunction with optimization modeling to design the next generation of diagnostic networks.

## Supplementary Information

**Additional file 1: Supplementary Figure S1.** Cumulative Number of Diagnostics with Additional Diseases. **Supplementary Table S1.** Definitions of disease levels (uncomplicated vs complicated). **Supplementary Table S2.** Mapping of medicines to disease levels. **Supplementary Table S3.** Mapping of diagnostics to disease levels. **Supplementary Table S4.** Mapping of diagnostics to diagnostic formats. **Supplementary Table S5.** Infrastructural limitations of diagnostic formats. **Supplementary Table S6.** Model output of diagnostics by health facility tier. **Supplementary Table S7.** Minimum equipment investment and workforce skill needs, by health system tier.

## Data Availability

The data underlying this article are available in the article, its online supplementary material, and previously published work.

## Abbreviations

ELISA: Enzyme-linked immunosorbent assay
G6PD: Glucose-6-phosphate-dehydrogenase deficiency
AFB: Acid-fast bacteria
NAAT: Nucleic acid amplification test
POC: Point-of-care
HPLC: High performance liquid chromatography
PALM: Pathology and Laboratory Medicine
EML: Essential Medicines List
NEML: National Essential Medicines List
EDL: Essential Diagnostics List
NEDL: National Essential Diagnostics List
GBD: Global Burden of Disease
LMICs: Low- and middle-income countries
UHC: Universal Health Coverage
