# Supplementary material for: Rational design of an essential diagnostics network to support Universal Health Coverage: a modeling analysis
Source: BMC Health Serv Res. 2022 Oct 1;22:1224. doi: 10.1186/s12913-022-08558-2 (PMC9526922; doi:10.1186/s12913-022-08558-2)
Supplement: Supplementary file 1 — Additional file 1: Supplementary Figure S1. Cumulative Number of Diagnostics with Additional Diseases. Supplementary Table S1. Definitions of disease levels (uncomplicated vs complicated). Supplementary Table S2. Mapping of medicines to disease levels. Supplementary Table S3. Mapping of diagnostics to disease levels. Supplementary Table S4. Mapping of diagnostics to diagnostic formats. Supplementary Table S5. Infrastructural limitations of diagnostic formats. Supplementary Table S6. Model output of diagnostics by health facility tier. Supplementary Table S7. Minimum equipment investment and workforce skill needs, by health system tier. [file 12913_2022_8558_MOESM1_ESM.pdf]

Supplementary Figure S1.

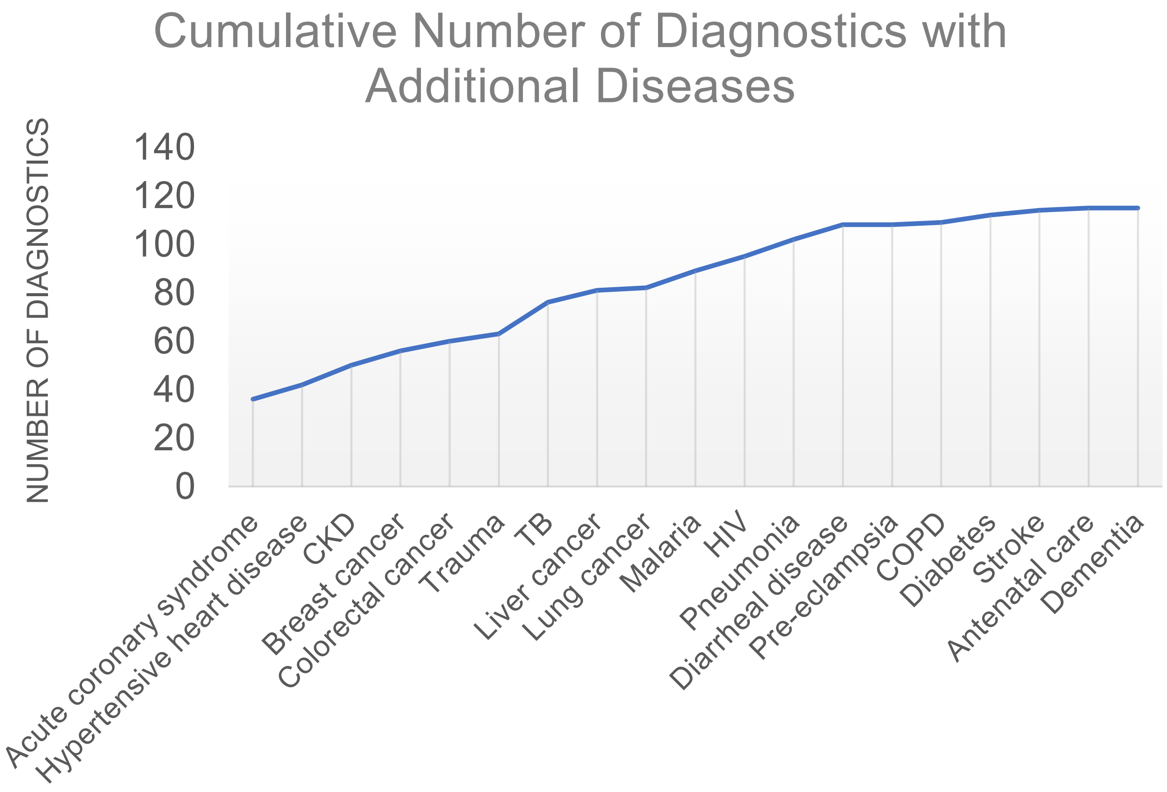

Supplementary Table S1. Definitions of disease levels.

Condition	Uncomplicated	Complicated
Antenatal care	n/a	n/a
Breast Cancer	resectable	non-resectable
Cerebrovascular disease	all else	any intervention indicated (thrombolysis, mechanical)
Chronic kidney disease	all else	volume overload, hyperkalemia, metabolic acidosis, and hyperphosphatemia, uremic encephalopathy, bleeding diathesis, dialysis, transplant
Colorectal cancer	resectable	non-resectable
COPD	all else	COPD exacerbation with hypoxemia requiring hospitalization
Dementia	all else	patients with an atypical syndrome (eg, younger patients [<60 years] or those with rapidly progressive dementia)
Diabetes mellitus	all else	ketoacidosis, hyperosmolar hyperglycemic state, end-organ disease (wounds, amputation, blindness, renal failure, macrovascular disease)
Diarrheal disease	all else	severe hypovolemia (altered consciousness, weak pulse); invasive diarrhea that do not respond to empiric antibiotic therapy; bacteremia; particular concern in HIV-infected individuals, hemolytic-uremic syndrome
HIV	CD4 > 200	CD4 < 200, aids-defining condition
Hypertensive heart disease	pharmacologic therapy, including common comorbidities	cardiac resynchronization therapy; advanced heart failure (episodes of refractory fluid retention, hypoperfusion, and/or recurrent malignant ventricular arrhythmias; evidence of severe cardiac dysfunction (on echocardiogram or right heart catheterization); and evidence of severe impairment of exercise capacity.)
Ischemic heart disease	all else	invasive reperfusion strategy, cardiogenic shock, left heart failure, sustained ventricular tachyarrhythmia.
Liver cancer	local	metastatic
Lower respiratory infections	Mild pneumonia (PSI < I-II)	moderate/severe pneumonia (PSI IV-V);
Lung cancer	resectable	non-resectable
Malaria	WHO non-severe malaria	WHO severe malaria (anemia, loss of consciousness, etc)
Preeclampsia, HELLP	pre-eclampsia without severe features	preeclampsia with severe features, HELLP, eclampsia
Trauma	Minor trauma according to a prehospital trauma triage scoring system	major trauma according to a prehospital trauma triage scoring system
Tuberculosis	all else	effusions, hemoptysis, pneumothorax, bronchiectasis, extensive pulmonary destruction (including pulmonary gangrene), fistula, tracheobronchial stenosis, malignancy, and chronic pulmonary aspergillosis

Supplementary Table S2. Mapping medicines to disease levels.

Condition	Condition Level	Medicine
Ischemic heart disease	triage	acetylsalicylic acid
	uncomplicated	clopidogrel
		simvastatin
	complicated	enoxaparin
		heparin sodium
		red blood cells
		streptokinase
		whole blood
Breast cancer	uncomplicated	anastrozole
		capecitabine
		carboplatin
		cyclophosphamide
		doxorubicin
		fluorouracil
		leuprorelin
		methotrexate
		paclitaxel
		tamoxifen
		trastuzumab
	complicated	filgrastim
		platelets
		red blood cells
		whole blood
Chronic kidney disease	uncomplicated	amlodipine
		bisoprolol
		enalapril
		furosemide
		hydrochlorothiazide
		losartan
		spironolactone
		verapamil
	complicated	red blood cells
		whole blood
Colorectal cancer	uncomplicated	capecitabine
		fluorouracil
		oxaliplatin
	complicated	filgrastim
		platelets
		red blood cells
		whole blood
COPD	uncomplicated	beclometasone
		budesonide_formoterol
		ipratropium bromide
		salbutamol
	complicated	amoxicillin
		amoxicillin_clavulanic acid

		cefalexin
		doxycycline
Diabetes	uncomplicated	gliclazide
		insulin injection (soluble)
		intermediate acting insulin
		metformin
	complicated	potassium chloride
Diarrheal disease	uncomplicated	ciprofloxacin
	complicated	azithromycin
		ciprofloxacin [PAR]
HIV	triage	condoms
	uncomplicated	dolutegravir
		efavirenz (EFV or EFZ)
		efavirenz_emtricitabine_tenofovir
		efavirenz_lamivudine_tenofovir
		emtricitabine_tenofovir
		zidovudine (ZDV or AZT)
	complicated	abacavir (ABC)
		abacavir (ABC)_lamivudine (3TC)
		atazanavir
		darunavir
		lopinavir_ritonavir (LPV/r)
		raltegravir
		zidovudine_lamivudine
Hypertensive heart disease	uncomplicated	amiloride
		bisoprolol
		furosemide
		hydrochlorothiazide
		losartan
		spironolactone
	complicated	digoxin
		dopamine
		red blood cells
		whole blood
Liver cancer	complicated	filgrastim
		platelets
		red blood cells
		sorafenib
		whole blood
Lung cancer	uncomplicated	carboplatin
		paclitaxel
	complicated	cisplatin
		etoposide
		filgrastim
		gefitinib
		gemcitabine
		platelets
		red blood cells
		vinorelbine

		whole blood
Malaria	uncomplicated	amodiaquine
		artemether
		artemether_lumefantrine
		artesunate
		artesunate _amodiaquine
		artesunate_mefloquine
		doxycycline
		primaquine
		sulfadoxine_pyrimethamine
	complicated	quinine
Lower respiratory infections	uncomplicated	amoxicillin
		amoxicillin_clavulanic acid
		ampicillin
		benzylpenicillin
		clarithromycin
		doxycycline
	complicated	aztreonam
		cefotaxime
		ceftriaxone
		cephalosporin5thGen
		gentamicin
		oxazolindinones
		phenoxymethylpenicillin
		tigecycline
Preeclampsia	complicated	hydralazine
		magnesium sulfate
		methyldopa
Cerebrovascular disease	uncomplicated	clopidogrel
	complicated	streptokinase
Trauma	complicated	dextran 70
		fresh frozen plasma
		halothane
		isoflurane
		ketamine
		oxygen
		platelets
		propofol
		red blood cells
		tranexamic acid
		whole blood
Tuberculosis	uncomplicated	ethambutol
		ethambutol_isoniazid
		ethambutol_isoniazid_ rifampicin
		isoniazid
		rifampicin_isoniazid
		rifampicin_isoniazid_pyrazinamide
		rifampicin_isoniazid_pyrazinamide_ethambutol
	complicated	amikacin

	bedaquiline
	capreomycin
	cycloserine
	delamanid
	ethionamide
	kanamycin
	levofloxacin
	linezolid
	moxifloxacin
	p aminosalicylic acid
	pyrazinamide
	rifabutin
	rifampicin
	rifapentine

Supplementary Table S3. Mapping diagnostics to diseases (condition- and medicine-related indications).

Condition	Condition Level	Diagnostic	Diagnostic Indication
Ischemic heart disease	triage	Cardiac marker	Diagnosis
	uncomplicated	CBC	Monitoring
			Toxicity
		Coagulation function (PT, PTT)	Pathophysiology
		Creatine kinase	Toxicity
		Creatinine clearance	Dosing/Safety
		CT scan	Complication
			DiffDiagnosis
		D-dimer products	DiffDiagnosis
		Echocardiogram	Diagnosis
		Electrolytes (Na, K, Cl)	Monitoring
		Glucose	CoMorbidity
		HbA1c	CoMorbidity
		Lipid panel	Pathophysiology
		Liver enzymes	Toxicity
		Liver function	Toxicity
		Magnesium	Monitoring
		Renal function	Complication
		X-ray	Diagnosis
			DiffDiagnosis
	complicated	anti-Xa assay (heparin)	Dosing/Safety
		B-type natriuretic peptide	Toxicity
		Basic interventional radiology	Diagnosis
		Blood gas	Toxicity
		CBC	Toxicity
		Coagulation function (PT, PTT)	Dosing/Safety
			Toxicity
		Creatinine clearance	Dosing/Safety
		CT scan	Diagnosis
		culture, Bacterial	Toxicity
		Direct antiglobulin test	Toxicity
		Fecal occult blood	Toxicity
		Haptoglobin	Toxicity
		Immunoglobulins, quantitative (IgG, IgA, IgM)	Toxicity
		Indirect antiglobulin test	Toxicity
		Lactate dehydrogenase	Toxicity
		Liver function	Toxicity
		Microscopy for microorganisms (gram, AFB, iodine, trichrome, india ink, KOH, etc.)	Toxicity
		Microscopy, CBC	Toxicity
		MRI	Diagnosis
		Nuclear radiology	Diagnosis
		PET scan	Diagnosis

		Red blood cell typing	Dosing/Safety
			Toxicity
		Urinalysis	Toxicity
Antenatal care	triage	Bacterial AST	Diagnosis
		Biochemical bacterial identfication	Diagnosis
		CBC	Diagnosis
		culture, Bacterial	Diagnosis
		Glucose	Diagnosis
		Hemoglobin	Diagnosis
		Microscopy for microorganisms (gram, AFB, iodine, trichrome, india ink, KOH, etc.)	Diagnosis
		Red blood cell typing	Diagnosis
		serology, HIV	Diagnosis
		serology, Syphilis (RPR, treponemal)	Diagnosis
		Ultrasound, POC	Diagnosis
		Urinalysis	Diagnosis
		Urinalysis, microscopic	Diagnosis
Breast cancer	triage	Mammography	Diagnosis
	uncomplicated	Advanced breast imaging	Diagnosis
		Alkaline phosphatase	Toxicity
		Calcium	Toxicity
		CBC	Toxicity
		Creatinine clearance	Dosing/Safety
		CT scan	Diagnosis
		Electrolytes (Na, K, Cl)	Toxicity
		Glucose	Toxicity
		HbA1c	Toxicity
		Histopathology/Cytopathology	Diagnosis
			DiffDiagnosis
		Human chorionic gonadotropin	Dosing/Safety
		Immunohistopathology/Immunocytopathology	Diagnosis
			DiffDiagnosis
			Pathophysiology
		Lipid panel	Toxicity
		Liver enzymes	Toxicity
		Liver function	Dosing/Safety
			Toxicity
		molecular, Breast cancer (ER, PR, HER2)	Pathophysiology
		molecular, Estrogen receptor	Companion
		molecular, HER2	Companion
		MRI	Diagnosis
		Nuclear radiology	Diagnosis
		PET scan	Diagnosis
		Phosphorus	Toxicity
		Renal function	Toxicity
		therapeutic drug monitoring, Methotrexate	Dosing/Safety

		Uric acid	Toxicity
		Urinalysis, microscopic	Toxicity
	complicated	B-type natriuretic peptide	Toxicity
		Blood gas	Toxicity
		CBC	Toxicity
		Coagulation function (PT, PTT)	Toxicity
		culture, Bacterial	Toxicity
		Direct antiglobulin test	Toxicity
		Haptoglobin	Toxicity
		Immunoglobulins, quantitative (IgG, IgA, IgM)	Toxicity
		Indirect antiglobulin test	Toxicity
		Lactate dehydrogenase	Toxicity
		Liver function	Toxicity
		Microscopy for microorganisms (gram, AFB, iodine, trichrome, india ink, KOH, etc.)	Toxicity
		Microscopy, CBC	Toxicity
		Red blood cell typing	Dosing/Safety
			Toxicity
		Urinalysis	Toxicity
COPD	uncomplicated	alpha-1 antitrypsin deficiency	DiffDiagnosis
		B-type natriuretic peptide	DiffDiagnosis
		Blood gas	Diagnosis
			Monitoring
		CBC	DiffDiagnosis
		Electrolytes (Na, K, Cl)	DiffDiagnosis
			Toxicity
		Glucose	DiffDiagnosis
		Renal function	Toxicity
		TSH	DiffDiagnosis
		X-ray	Diagnosis
	complicated	Bacterial AST	Complication
		Biochemical bacterial identfication	Complication
		Cardiac marker	DiffDiagnosis
		CBC	Toxicity
		Creatinine clearance	Dosing/Safety
		CT scan	Complication
			DiffDiagnosis
		culture, Bacterial	Complication
		Liver enzymes	Toxicity
		Liver function	Toxicity
		Microscopy for microorganisms (gram, AFB, iodine, trichrome, india ink, KOH, etc.)	Complication
		nucleic acid testing, Tuberculosis	CoMorbidity
		Renal function	Toxicity
Chronic kidney disease	triage	Renal function	Diagnosis
			Monitoring

	Urinalysis	Diagnosis
		Monitoring
uncomplicated	Bicarbonate	Toxicity
	Calcium	Monitoring
		Toxicity
	CBC	Toxicity
	Creatinine clearance	Dosing/Safety
	Electrolytes (Na, K, Cl)	Monitoring
		Toxicity
	Glucose	Toxicity
	Hemoglobin	Complication
	Human chorionic gonadotropin	Dosing/Safety
	Lipid panel	CoMorbidity
	Liver enzymes	Toxicity
	Liver function	Dosing/Safety
		Toxicity
	Magnesium	Toxicity
	Phosphorus	Monitoring
	Renal function	Toxicity
	Ultrasound	Diagnosis
	Uric acid	Toxicity
	Urinalysis, microscopic	Diagnosis
	Urine albumin/creatinine	Diagnosis
		Monitoring
	Urine protein/creatinine	Diagnosis
		Monitoring
complicated	B-type natriuretic peptide	Toxicity
	Blood gas	Complication
		Toxicity
	CBC	Toxicity
	Coagulation function (PT, PTT)	Toxicity
	culture, Bacterial	Toxicity
	Direct antiglobulin test	Toxicity
	Haptoglobin	Toxicity
	Immunoglobulins, quantitative (IgG, IgA, IgM)	Toxicity
	Immunohistopathology/Immunocytopathology	Diagnosis
	Indirect antiglobulin test	Toxicity
	Lactate dehydrogenase	Toxicity
	Liver function	Toxicity
	Microscopy for microorganisms (gram, AFB, iodine, trichrome, india ink, KOH, etc.)	Toxicity
	Microscopy, CBC	Toxicity
	Parathyroid hormone	Complication
	Red blood cell typing	Dosing/Safety
		Toxicity

		Urinalysis	Toxicity
		Vitamin D, hydroxy (25)	Complication
Colorectal cancer	triage	Fecal Immunochemical Test	Diagnosis
		Fecal occult blood	Diagnosis
		Hemoglobin	Diagnosis
	uncomplicated	Carcinoembryonic antigen (CEA)	Monitoring
		CBC	Toxicity
		Coagulation function (PT, PTT)	Toxicity
		Creatinine clearance	Dosing/Safety
		CT scan	Diagnosis
		Electrolytes (Na, K, Cl)	Toxicity
		Glucose	Toxicity
		Histopathology/Cytopathology	Diagnosis
			DiffDiagnosis
		Human chorionic gonadotropin	Dosing/Safety
		Immunohistopathology/Immunocytopathology	Diagnosis
			DiffDiagnosis
			Pathophysiology
		Iron deficiency panel	CoMorbidity
		Liver enzymes	Toxicity
		Liver function	Dosing/Safety
			Toxicity
		Magnesium	Toxicity
		Microsatellite instability testing	Pathophysiology
		MRI	Diagnosis
		Renal function	Toxicity
		Ultrasound	Diagnosis
	complicated	B-type natriuretic peptide	Toxicity
		Blood gas	Toxicity
		CBC	Toxicity
		Coagulation function (PT, PTT)	Toxicity
		culture, Bacterial	Toxicity
		Direct antiglobulin test	Toxicity
		Haptoglobin	Toxicity
		Immunoglobulins, quantitative (IgG, IgA, IgM)	Toxicity
		Indirect antiglobulin test	Toxicity
		Lactate dehydrogenase	Toxicity
		Liver function	Toxicity
		Microscopy for microorganisms (gram, AFB, iodine, trichrome, india ink, KOH, etc.)	Toxicity
		Microscopy, CBC	Toxicity
		PET scan	Diagnosis
		Red blood cell typing	Dosing/Safety
			Toxicity
		Urinalysis	Toxicity
Dementia	uncomplicated	CBC	DiffDiagnosis
		Hemoglobin	DiffDiagnosis
		serology, Syphilis (RPR, treponemal)	DiffDiagnosis
		TSH	DiffDiagnosis

		Vitamin B12	DiffDiagnosis
	complicated	CT scan	Diagnosis
		MRI	Diagnosis
Diabetes	triage	Glucose	Diagnosis
			Monitoring
		Urinalysis	Diagnosis
	uncomplicated	CBC	Toxicity
		Creatinine clearance	Dosing/Safety
		Electrolytes (Na, K, Cl)	Complication
			Toxicity
		Glucose	Toxicity
		HbA1c	Diagnosis
			Monitoring
		Lipid panel	CoMorbidity
		Phosphorus	Complication
		Renal function	Complication
			Monitoring
		Urine albumin/creatinine	Monitoring
		Vitamin B12	Toxicity
	complicated	Blood gas	Complication
			Toxicity
		Electrolytes (Na, K, Cl)	Dosing/Safety
		Ketones	Complication
		Renal function	Toxicity
Diarrheal disease	triage	Fecal leukocytes	Diagnosis
			Monitoring
		Microscopy, ova and parasites	Diagnosis
			Monitoring
	uncomplicated	CBC	Toxicity
		Creatinine clearance	Dosing/Safety
		G6PD enzyme testing	Dosing/Safety
		Liver enzymes	Toxicity
		Renal function	Toxicity
	complicated	antigen, Entamoeba	DiffDiagnosis
		antigen, Shiga	Diagnosis
		Bacterial AST	DST
		Biochemical bacterial identfication	Diagnosis
		CBC	Complication
			Toxicity
		Creatinine clearance	Dosing/Safety
		culture, Bacterial	Diagnosis
		G6PD enzyme testing	Toxicity
		Hemoglobin	Complication
		Liver enzymes	Toxicity
		Microscopy for microorganisms (gram, AFB, iodine, trichrome, india ink, KOH, etc.)	Diagnosis
		nucleic acid testing, Shiga	Diagnosis
		Renal function	Toxicity
		serology, Entamoeba	DiffDiagnosis

		serology, Typhoid fever	Diagnosis
HIV	triage	serology, HIV	Diagnosis
	uncomplicated	Amylase	Toxicity
		Antinuclear antibodies	DiffDiagnosis
		CBC	Complication
			Toxicity
		Creatinine clearance	Dosing/Safety
		Electrolytes (Na, K, Cl)	Toxicity
		Glucose	Toxicity
		HbA1c	Toxicity
		HIV RNA quantitative	Monitoring
		Human chorionic gonadotropin	Dosing/Safety
			Monitoring
		Lipase	Toxicity
		Lipid panel	Toxicity
		Liver enzymes	Toxicity
		Liver function	Toxicity
		Lymphocyte CD4	Monitoring
		nucleic acid testing, Chlamydia	CoMorbidity
		nucleic acid testing, N. gonorrhoeae	CoMorbidity
		Phosphorus	Toxicity
		Qualitative HIV virological (RNA, DNA, or US p24 Ag)	Diagnosis
		Renal function	Complication
			Toxicity
		serology, HBV	CoMorbidity
		serology, HCV	CoMorbidity
		serology, Syphilis (RPR, treponemal)	CoMorbidity
		Urinalysis	Complication
	complicated	Amylase	Toxicity
		CBC	Toxicity
		Creatinine clearance	Dosing/Safety
		Electrolytes (Na, K, Cl)	Toxicity
		Glucose	Toxicity
		HbA1c	Toxicity
		Human chorionic gonadotropin	Dosing/Safety
		Lipase	Toxicity
		Lipid panel	Toxicity
		Liver enzymes	Toxicity
		Liver function	Dosing/Safety
			Toxicity
		Renal function	Toxicity
Hypertensive heart disease	triage	Glucose	CoMorbidity
		HbA1c	CoMorbidity
		Lipid panel	Pathophysiology

uncomplicated	B-type natriuretic peptide	Diagnosis
		Monitoring
	Bicarbonate	Toxicity
	Calcium	Toxicity
	CBC	Toxicity
	Creatinine clearance	Dosing/Safety
	CT scan	Monitoring
	Echocardiogram	Diagnosis
	Electrolytes (Na, K, Cl)	Complication
		Toxicity
	Glucose	Toxicity
	Hemoglobin	Monitoring
	Human chorionic gonadotropin	Dosing/Safety
	Liver enzymes	Complication
		Toxicity
	Liver function	Complication
		Toxicity
	Magnesium	Toxicity
	Renal function	Complication
		Toxicity
	Uric acid	Toxicity
	X-ray	Diagnosis
complicated	B-type natriuretic peptide	Toxicity
	Basic interventional radiology	Diagnosis
	Blood gas	Toxicity
	Calcium	Toxicity
	CBC	Toxicity
	Coagulation function (PT, PTT)	Toxicity
	Creatinine clearance	Dosing/Safety
	CT scan	Diagnosis
	culture, Bacterial	Toxicity
	Direct antiglobulin test	Toxicity
	Electrolytes (Na, K, Cl)	Toxicity
	Haptoglobin	Toxicity
	Immunoglobulins, quantitative (IgG, IgA, IgM)	Toxicity
	Indirect antiglobulin test	Toxicity
	Lactate dehydrogenase	Toxicity
	Liver function	Toxicity
	Magnesium	Toxicity
	Microscopy for microorganisms (gram, AFB, iodine, trichrome, india ink, KOH, etc.)	Toxicity
	Microscopy, CBC	Toxicity
	MRI	Diagnosis
	PET scan	Diagnosis
	Red blood cell typing	Dosing/Safety
		Toxicity

		Renal function	Toxicity
		Urinalysis	Toxicity
Liver cancer	uncomplicated	Alkaline phosphatase	Diagnosis
		Alpha-fetoprotein	Diagnosis
			Monitoring
		CA 19-9	DiffDiagnosis
		Carcinoembryonic antigen (CEA)	Diagnosis
			DiffDiagnosis
		CBC	Diagnosis
		Coagulation function (PT, PTT)	Diagnosis
		CT scan	Diagnosis
		Histopathology/Cytopathology	Diagnosis
			DiffDiagnosis
		Immunohistopathology/Immunocytopathology	DiffDiagnosis
		Liver enzymes	Diagnosis
		Liver function	Diagnosis
		MRI	Diagnosis
		Renal function	Diagnosis
		serology, HBV	Pathophysiology
		serology, HCV	Pathophysiology
		Ultrasound	Diagnosis
	complicated	B-type natriuretic peptide	Toxicity
		Blood gas	Toxicity
		Calcium	Toxicity
		CBC	Toxicity
		Coagulation function (PT, PTT)	Dosing/Safety
			Toxicity
		Creatinine clearance	Dosing/Safety
		culture, Bacterial	Toxicity
		Direct antiglobulin test	Toxicity
		Electrolytes (Na, K, Cl)	Toxicity
		Haptoglobin	Toxicity
		Immunoglobulins, quantitative (IgG, IgA, IgM)	Toxicity
		Indirect antiglobulin test	Toxicity
		Lactate dehydrogenase	Toxicity
		Liver function	Dosing/Safety
			Toxicity
		Magnesium	Toxicity
		Microscopy for microorganisms (gram, AFB, iodine, trichrome, india ink, KOH, etc.)	Toxicity
		Microscopy, CBC	Toxicity
		Red blood cell typing	Dosing/Safety
			Toxicity
		Urinalysis	Toxicity
Lung cancer	triage	X-ray	Diagnosis
	uncomplicated	CBC	Toxicity
		Creatinine clearance	Dosing/Safety
		Histopathology/Cytopathology	Diagnosis
			DiffDiagnosis

		Immunohistopathology/Immunocytopathology	Diagnosis
			DiffDiagnosis
			Pathophysiology
		Liver function	Dosing/Safety
		MRI	Diagnosis
		Nuclear radiology	Diagnosis
	complicated	B-type natriuretic peptide	Toxicity
		Blood gas	Toxicity
		Calcium	Toxicity
		CBC	Toxicity
		Coagulation function (PT, PTT)	Toxicity
		Creatinine clearance	Dosing/Safety
		CT scan	Diagnosis
		culture, Bacterial	Toxicity
		Direct antiglobulin test	Toxicity
		Electrolytes (Na, K, Cl)	Toxicity
		Haptoglobin	Toxicity
		Immunoglobulins, quantitative (IgG, IgA, IgM)	Toxicity
		Indirect antiglobulin test	Toxicity
		Lactate dehydrogenase	Toxicity
		Liver enzymes	Toxicity
		Liver function	Dosing/Safety
			Toxicity
		Magnesium	Toxicity
		Microscopy for microorganisms (gram, AFB, iodine, trichrome, india ink, KOH, etc.)	Toxicity
		Microscopy, CBC	Toxicity
		molecular, Epidermal Growth Factor Receptor	Companion
		PET scan	Diagnosis
		Red blood cell typing	Dosing/Safety
			Toxicity
		Renal function	Toxicity
		Urinalysis	Toxicity
Malaria	triage	antigen, Malaria	Diagnosis
		Microscopy, Malaria	Diagnosis
	uncomplicated	CBC	Complication
			Toxicity
		Cooximetry	Toxicity
		Electrolytes (Na, K, Cl)	Toxicity
		G6PD enzyme testing	Dosing/Safety
		Glucose	Complication
		Haptoglobin	Toxicity
		Human chorionic gonadotropin	Dosing/Safety
		Lactate dehydrogenase	Toxicity
		Liver enzymes	Toxicity

		Liver function	Complication
			Toxicity
		Microscopy, Malaria	Monitoring
		Renal function	Complication
			Toxicity
		serology, Typhoid fever	DiffDiagnosis
		Urinalysis	Complication
			Dosing/Safety
			Toxicity
		X-ray	DiffDiagnosis
	complicated	Blood gas	Complication
		CBC	Toxicity
		Coagulation function (PT, PTT)	Toxicity
		Creatinine clearance	Dosing/Safety
		CSF analysis (microscopy, cell counts, glucose, protein)	DiffDiagnosis
		culture, Bacterial	CoMorbidity
			DiffDiagnosis
		Glucose	Toxicity
		Liver function	Toxicity
		MRI	Complication
Lower respiratory infections	uncomplicated	CBC	Toxicity
		Creatinine clearance	Dosing/Safety
		Electrolytes (Na, K, Cl)	Toxicity
		ESR/CRP	Diagnosis
		Liver enzymes	Toxicity
		Liver function	Toxicity
		Renal function	Toxicity
		X-ray	Diagnosis
	complicated	antigen, Legionella (urine)	Diagnosis
		antigen, Pneumococcus (urine)	Diagnosis
		B-type natriuretic peptide	DiffDiagnosis
		Bacterial AST	DST
		Biochemical bacterial identfication	Diagnosis
		CBC	Toxicity
		Coagulation function (PT, PTT)	Toxicity
		Creatinine clearance	Dosing/Safety
		CT scan	Diagnosis

		culture, Bacterial	Diagnosis
		D-dimer products	DiffDiagnosis
		Direct antiglobulin test	Toxicity
		Liver enzymes	Toxicity
		Liver function	Dosing/Safety
			Toxicity
		Microscopy for microorganisms (gram, AFB, iodine, trichrome, india ink, KOH, etc.)	Diagnosis
		nucleic acid testing, Respiratory virus panel	Diagnosis
		Renal function	Toxicity
		therapeutic drug monitoring, Gentamicin	Dosing/Safety
		Urinalysis, microscopic	Toxicity
Preeclampsia	triage	Urinalysis	Diagnosis
			Monitoring
	uncomplicated	Antinuclear antibodies	DiffDiagnosis
		CBC	Diagnosis
			Monitoring
		Haptoglobin	Diagnosis
			Monitoring
		Hemoglobin	Diagnosis
			Monitoring
		Lactate dehydrogenase	Diagnosis
			Monitoring
		Liver enzymes	Diagnosis
			Monitoring
		Microscopy, CBC	Diagnosis
			Monitoring
		Renal function	Monitoring
		Urine protein/creatinine	Diagnosis
	complicated	Antinuclear antibodies	Toxicity
		CBC	Toxicity
		Coagulation function (PT, PTT)	DiffDiagnosis
		Creatinine clearance	Dosing/Safety
		D-dimer products	DiffDiagnosis
		Direct antiglobulin test	Toxicity
		Fibrinogen	DiffDiagnosis
		Indirect antiglobulin test	Toxicity
		Liver enzymes	Toxicity
		Liver function	Toxicity
		Magnesium	Dosing/Safety
Cerebrovascular disease	uncomplicated	Antiphospholipid antibodies	Pathophysiology
		Basic interventional radiology	Diagnosis
		CBC	Pathophysiology
			Toxicity
		Coagulation function (PT, PTT)	Pathophysiology
		Complex interventional radiology	Diagnosis

		CT scan	Diagnosis
		culture, Bacterial	DiffDiagnosis
		D-dimer products	DiffDiagnosis
		Electrolytes (Na, K, Cl)	Monitoring
		Glucose	DiffDiagnosis
		Lipid panel	Pathophysiology
		MRI	Diagnosis
		Renal function	Monitoring
		Ultrasound	Diagnosis
	complicated	CBC	Toxicity
		Coagulation function (PT, PTT)	Toxicity
Trauma	uncomplicated	Creatine kinase	Complication
		CT scan	Diagnosis
		Glucose	Complication
		Human chorionic gonadotropin	CoMorbidity
		Ultrasound	Diagnosis
		X-ray	Diagnosis
	complicated	B-type natriuretic peptide	Toxicity
		Blood gas	Complication
			Dosing/Safety
			Monitoring
			Toxicity
		CBC	Toxicity
		Coagulation function (PT, PTT)	Complication
			Toxicity
		Creatine kinase	Toxicity
		Creatinine clearance	Dosing/Safety
		culture, Bacterial	Toxicity
		Direct antiglobulin test	Toxicity
		Electrolytes (Na, K, Cl)	Toxicity
		Fibrinogen	Toxicity
		Fluoroscopy	Diagnosis
		Haptoglobin	Toxicity
		Hemoglobin	Complication
		Immunoglobulins, quantitative (IgG, IgA, IgM)	Toxicity
		Indirect antiglobulin test	Toxicity
		Lactate	Monitoring
		Lactate dehydrogenase	Toxicity
		Liver enzymes	Toxicity
		Liver function	Toxicity
		Microscopy for microorganisms (gram, AFB, iodine, trichrome, india ink, KOH, etc.)	Toxicity
		Microscopy, CBC	Toxicity

		MRI	Diagnosis
		Red blood cell typing	Complication
			Dosing/Safety
			Toxicity
		Renal function	Toxicity
		Urinalysis	Toxicity
Tuberculosis	triage	Microscopy for microorganisms (gram, AFB, iodine, trichrome, india ink, KOH, etc.)	Diagnosis
		nucleic acid testing, Tuberculosis	Diagnosis
		X-ray	Diagnosis
	uncomplicated	CBC	Toxicity
		Creatinine clearance	Dosing/Safety
		culture, Tuberculosis	Diagnosis
			Monitoring
		G6PD enzyme testing	Dosing/Safety
		Liver enzymes	Toxicity
		Lymphocyte CD4	Pathophysiology
		Microscopy for microorganisms (gram, AFB, iodine, trichrome, india ink, KOH, etc.)	Monitoring
		nucleic acid testing, Tuberculosis	DST
		serology, HIV	Pathophysiology
		TB AST	DST
		TB Line probe assays for INH, RIF, 2nd line agents (AST)	DST
		TB Lipoarabinomannan, urine (LF-LAM)	Diagnosis
		Uric acid	Toxicity
		X-ray	Monitoring
	complicated	Amylase	Toxicity
		Calcium	Toxicity
		CBC	Toxicity
		Creatinine clearance	Dosing/Safety
		CSF analysis (microscopy, cell counts, glucose, protein)	Diagnosis
		culture, Bacterial	DiffDiagnosis
		culture, Fungal	DiffDiagnosis
		Electrolytes (Na, K, Cl)	Toxicity
		G6PD enzyme testing	Dosing/Safety
		Glucose	Toxicity
		Histopathology/Cytopathology	DiffDiagnosis
		Human chorionic gonadotropin	Dosing/Safety
		Lactate	Toxicity
		Lipase	Toxicity
		Liver enzymes	Toxicity
		Liver function	Dosing/Safety
			Toxicity
		Magnesium	Toxicity
		Renal function	Toxicity

	therapeutic drug monitoring, Amikacin	Dosing/Safety
	TSH	Toxicity
	Uric acid	Toxicity

Supplementary Table S4. Mapping of diagnostics to diagnostic formats.

Diagnostic	Diagnostic Format
Advanced breast imaging	Advanced breast imaging
Alkaline phosphatase	Automated chemistry analyzer
alpha-1 antitrypsin deficiency	Automated chemistry analyzer
Alpha-fetoprotein	Automated immunoassay analyzer
Amylase	Automated chemistry analyzer
anti-Xa assay (heparin)	Automated coagulation analyzer
antigen, Entamoeba	Automated immunoassay analyzer
antigen, Legionella (urine)	Automated immunoassay analyzer
	RDT
antigen, Malaria	RDT
antigen, Pneumococcus (urine)	RDT
antigen, Shiga	Automated immunoassay analyzer
	RDT
Antinuclear antibodies	Indirect immunofluorescence
Antiphospholipid antibodies	Automated coagulation analyzer
B-type natriuretic peptide	Automated immunoassay analyzer
Bacterial AST	Antimicrobial susceptibility testing
Basic interventional radiology	Interventional radiology, basic
Bicarbonate	Benchtop/handheld analyzer
Biochemical bacterial identfication	Automated biochemical microbial identification
Blood gas	Benchtop/handheld analyzer
CA 19-9	Automated immunoassay analyzer
Calcium	Automated chemistry analyzer
Carcinoembryonic antigen (CEA)	Automated immunoassay analyzer
Cardiac marker	Automated immunoassay analyzer
	Benchtop/handheld analyzer
CBC	Automated hematology analyzer
Coagulation function (PT, PTT)	Automated coagulation analyzer
	Benchtop/handheld analyzer
Complex interventional radiology	Interventional radiology, complex
Cooximetry	Benchtop/handheld analyzer
Creatine kinase	Automated chemistry analyzer
Creatinine clearance	Automated chemistry analyzer
CSF analysis (microscopy, cell counts, glucose, protein)	Automated chemistry analyzer
	Automated hematology analyzer
	Hemocytometer
CT scan	CT scan
culture, Bacterial	Culture
culture, Fungal	Culture
culture, Tuberculosis	TB Culture
D-dimer products	Automated immunoassay analyzer
Direct antiglobulin test	Red blood cell agglutination
Echocardiogram	Echocardiogram
Electrolytes (Na, K, Cl)	Automated chemistry analyzer
ESR/CRP	Automated immunoassay analyzer
	RDT
Fecal Immunochemical Test	RDT
Fecal leukocytes	Microscopy, with stains
Fecal occult blood	RDT
Fibrinogen	Automated coagulation analyzer
	Benchtop/handheld analyzer

Fluoroscopy	Fluoroscopy
G6PD enzyme testing	RDT
	Semi-quantitative fluorescent spot test
Glucose	Automated chemistry analyzer
	Glucometer
Haptoglobin	Automated chemistry analyzer
HbA1c	Benchtop/handheld analyzer
	High performance liquid chromatography
Hemoglobin	Hemoglobinometer
Histopathology/Cytopathology	Microscopy, with stains
HIV RNA quantitative	Automated nucleic acid analyzer
	Benchtop/handheld analyzer
Human chorionic gonadotropin	Automated immunoassay analyzer
	RDT
Immunoglobulins, quantitative (IgG, IgA, IgM)	Automated immunoassay analyzer
Immunohistopathology/Immunocytopathology	Microscopy, with immunohistochemistry
Indirect antiglobulin test	Red blood cell agglutination
Iron deficiency panel	Automated immunoassay analyzer
Ketones	Automated immunoassay analyzer
Lactate	Benchtop/handheld analyzer
Lactate dehydrogenase	Automated chemistry analyzer
Lipase	Automated chemistry analyzer
Lipid panel	Automated chemistry analyzer
Liver enzymes	Automated chemistry analyzer
Liver function	Automated chemistry analyzer
Lymphocyte CD4	Benchtop/handheld analyzer
	Flow cytometry
Magnesium	Automated chemistry analyzer
Mammography	Mammography
Microsatellite instability testing	Automated nucleic acid analyzer
Microscopy for microorganisms (gram, AFB, iodine, trichrome, india ink, KOH, etc.)	Microscopy, with stains
Microscopy, CBC	Hematology smear microscopy
Microscopy, Malaria	Malaria smear microscopy
Microscopy, ova and parasites	Stool microscopy
molecular, Breast cancer (ER, PR, HER2)	Automated nucleic acid analyzer
molecular, Epidermal Growth Factor Receptor	Automated nucleic acid analyzer
molecular, Estrogen receptor	Benchtop/handheld analyzer
	Microscopy, with immunohistochemistry
molecular, HER2	Benchtop/handheld analyzer
	Microscopy, with immunohistochemistry
MRI	MRI
Nuclear radiology	Nuclear radiology
nucleic acid testing, Chlamydia	Automated nucleic acid analyzer
nucleic acid testing, N. gonorrhoeae	Automated nucleic acid analyzer
nucleic acid testing, Respiratory virus panel	Automated nucleic acid analyzer
nucleic acid testing, Shiga	Automated nucleic acid analyzer
nucleic acid testing, Tuberculosis	Automated nucleic acid analyzer
	Benchtop/handheld analyzer
Parathyroid hormone	Automated immunoassay analyzer
PET scan	PET scan
Phosphorus	Automated chemistry analyzer
Qualitative HIV virological (RNA, DNA, or US p24 Ag)	Automated nucleic acid analyzer
	Benchtop/handheld analyzer
Red blood cell typing	Slide agglutination

Renal function	Automated chemistry analyzer
serology, Entamoeba	Automated immunoassay analyzer
serology, HBV	Automated immunoassay analyzer
serology, HCV	Automated immunoassay analyzer
	RDT
serology, HIV	Automated immunoassay analyzer
	RDT
serology, Syphilis (RPR, treponemal)	Automated immunoassay analyzer
	RDT
serology, Typhoid fever	ELISA
TB AST	Antimicrobial susceptibility testing, TB
TB Line probe assays for INH, RIF, 2nd line agents (AST)	Antimicrobial susceptibility testing, TB
TB Lipoarabinomannan, urine (LF-LAM)	RDT
therapeutic drug monitoring, Amikacin	Automated chemistry analyzer
therapeutic drug monitoring, Gentamicin	Automated chemistry analyzer
therapeutic drug monitoring, Methotrexate	Automated chemistry analyzer
TSH	Automated immunoassay analyzer
Ultrasound	Ultrasound
Ultrasound, POC	Ultrasound, POC
Uric acid	Automated chemistry analyzer
Urinalysis	Urine Dipstick
Urinalysis, microscopic	Urinalysis, microscopic
Urine albumin/creatinine	Automated chemistry analyzer
Urine protein/creatinine	Automated chemistry analyzer
Vitamin B12	Automated chemistry analyzer
Vitamin D, hydroxy (25)	Automated immunoassay analyzer
X-ray	X-ray

Supplementary Table S5. Infrastructural limitations of diagnostic formats.

Diagnostic Format	Tier
Advanced breast imaging	Tertiary
Antimicrobial susceptibility testing	Secondary
Antimicrobial susceptibility testing, TB	Tertiary
Automated biochemical microbial identification	Secondary
Automated chemistry analyzer	Secondary
Automated coagulation analyzer	Secondary
Automated hematology analyzer	Secondary
Automated immunoassay analyzer	Secondary
Automated nucleic acid analyzer	Tertiary
Benchtop/handheld analyzer	Secondary
CT scan	Tertiary
Culture	Secondary
ELISA	Secondary
Echocardiogram	Tertiary
Flow cytometry	Tertiary
Fluoroscopy	Tertiary
Glucometer	Primary
Hematology smear microscopy	Secondary
Hemocytometer	Secondary
Hemoglobinometer	Primary
High performance liquid chromatography	Tertiary
Indirect immunofluorescence	Tertiary
Interventional radiology, basic	Tertiary
Interventional radiology, complex	Tertiary
MRI	Tertiary
Malaria smear microscopy	Primary
Mammography	Tertiary
Microscopy, with immunohistochemistry	Tertiary
Microscopy, with stains	Secondary
No instrument	Primary
Nuclear radiology	Tertiary
PET scan	Tertiary
RDT	Primary
Red blood cell agglutination	Secondary
Semi-quantitative fluorescent spot test	Tertiary
Slide agglutination	Secondary
Stool microscopy	Primary
TB Culture	Tertiary
Ultrasound	Secondary
Ultrasound, POC	Primary
Urinalysis, microscopic	Primary
Urine Dipstick	Primary
X-ray	Secondary

Supplementary Table S6. Model output of diagnostics by health facility tier.

Service	Primary	Secondary	Tertiary
Chemistry	RDT: Human chorionic gonadotropin; ESR/CRP; Fecal Immunochemical Test; Fecal occult blood<br><br>Glucometer: Glucose	Automated chemistry analyzer: alpha-1 antitrypsin deficiency; Amylase*; Liver function*; Calcium; Creatine kinase; Creatinine clearance*; Electrolytes (Na, K, Cl)*; Glucose*; Haptoglobin*; Lactate dehydrogenase*; Lipase*; Lipid panel*; Liver enzymes*; Magnesium; Phosphorus*; Renal function*; Uric acid*; Urine albumin/creatinine; Urine protein/creatinine; Vitamin B12<br><br>Automated immunoassay analyzer: B-type natriuretic peptide; Human chorionic gonadotropin*; ESR/CRP*; Cardiac marker; TSH<br><br>Benchtop/handheld analyzer: Bicarbonate; Blood gas; Cooximetry*; HbA1c*; Cardiac marker	Automated immunoassay analyzer: Vitamin D, hydroxy (25); Alpha-fetoprotein; CA 19-9; Carcinoembryonic antigen (CEA); Iron deficiency panel; Ketones; Parathyroid hormone<br><br>Automated chemistry analyzer: Alkaline phosphatase<br><br>High performance liquid chromatography: HbA1c**<br><br>Benchtop/handheld analyzer: Lactate
Hematology	RDT: G6PD enzyme testing<br><br>Hemoglobinometer: Hemoglobin<br><br>Urine Dipstick: Urinalysis<br><br>Urinalysis, microscopic: Urinalysis, microscopic	Automated hematology analyzer: CBC*<br><br>Microscopy, with stains: Fecal leukocytes*<br><br>Hematology smear microscopy: Microscopy, CBC	Automated chemistry analyzer: CSF analysis (microscopy, cell counts, glucose, protein)<br><br>Automated hematology analyzer: CSF analysis (microscopy, cell counts, glucose, protein)<br><br>Hemocytometer: CSF analysis (microscopy, cell counts, glucose, protein)<br><br>Semi-quantitative fluorescent spot test: G6PD enzyme testing**

Microbiology	RDT: antigen, Malaria; serology, HCV; serology, HIV; serology, Syphilis (RPR, treponemal); TB Lipoarabinomannan, urine (LF-LAM)<br><br>Microscopy, Malaria<br><br>Microscopy, ova and parasites	Biochemical bacterial identfication*<br><br>culture, Bacterial*<br><br>Bacterial antimicrobial susceptibility testing*<br><br>Microscopy, with stains: Microscopy for microorganisms (gram, AFB, iodine, trichrome, india ink, KOH, etc.)*<br><br>Benchtop/handheld analyzer: Qualitative HIV virological (RNA, DNA, or US p24 Ag)*; HIV RNA quantitative*; nucleic acid testing, Tuberculosis*<br><br>Automated immunoassay analyzer: serology, HBV*; serology, HCV*; serology, HIV*; serology, Syphilis (RPR, treponemal)*<br><br>ELISA: serology, Typhoid fever*	Automated immunoassay analyzer: antigen, Entamoeba; antigen, Legionella (urine); antigen, Shiga; serology, Entamoeba<br><br>RDT: antigen, Legionella (urine); antigen, Pneumococcus (urine); antigen, Shiga<br><br>culture, Fungal<br><br>culture, Tuberculosis**<br><br>Automated nucleic acid analyzer: nucleic acid testing, Chlamydia**; Qualitative HIV virological (RNA, DNA, or US p24 Ag)**; HIV RNA quantitative**; nucleic acid testing, N. gonorrhoeae**; nucleic acid testing, Respiratory virus panel; nucleic acid testing, Shiga; nucleic acid testing, Tuberculosis**<br><br>Antimicrobial susceptibility testing, TB**
Radiology	Ultrasound, POC	Ultrasound<br><br>X-ray*	Advanced breast imaging<br><br>CT scan*<br><br>Echocardiogram*<br><br>Fluoroscopy<br><br>Basic interventional radiology*<br><br>Complex interventional radiology*<br><br>Mammography**<br><br>MRI*<br><br>Nuclear radiology<br><br>PET scan
Blood bank	NA	Slide agglutination: Red blood cell typing*	Red blood cell agglutination: Direct antiglobulin test; Indirect antiglobulin test
Coagulation	NA	Automated coagulation analyzer: Antiphospholipid antibodies; Coagulation function (PT, PTT)<br><br>Benchtop/handheld analyzer: Coagulation function (PT, PTT)<br><br>Automated immunoassay analyzer: D-dimer products	Automated coagulation analyzer: anti-Xa assay (heparin); Fibrinogen<br><br>Benchtop/handheld analyzer: Fibrinogen
Flow cytometry	NA	Benchtop/handheld analyzer: Lymphocyte CD4*	Flow cytometry: Lymphocyte CD4**

Histopathology	NA	NA	Microscopy, with immunohistochemistry: Immunohistopathology/Immunocytopathology<br><br>Microscopy, with stains: Histopathology/Cytopathology
Immunology	NA	NA	Indirect immunofluorescence: Antinuclear antibodies**<br><br>Automated immunoassay analyzer: Immunoglobulins, quantitative (IgG, IgA, IgM)
Molecular (non-Microbiology)	NA	NA	Benchtop/handheld analyzer: molecular, Estrogen receptor; molecular, HER2<br><br>Microscopy, with immunohistochemistry: molecular, Estrogen receptor; molecular, HER2<br><br>Automated nucleic acid analyzer: Microsatellite instability testing; molecular, Breast cancer (ER, PR, HER2); molecular, Epidermal Growth Factor Receptor
Toxicology	NA	NA	Automated chemistry analyzer: therapeutic drug monitoring, Amikacin; therapeutic drug monitoring, Gentamicin; therapeutic drug monitoring, Methotrexate

* Diagnostic should be placed 1 tier lower but logistical constraints preclude this option. Requests from the lower tier (specimens or referrals) should be performed at this level.
** Diagnostic should be placed 2 tiers lower but logistical constraints preclude this option. Requests from the lower tiers (specimens or referrals) should be performed at this level.

Note: all diagnostics available at lower tier levels are also available at higher tier levels but are unlisted in this table. Tests may be performed in different formats at different tiers or even within the same tier, and placement of all formats may not be necessary.

Supplementary Table S7. Minimum equipment investment and workforce skill needs, by health system tier.

Equipment needs	Minimum workforce needs
Primary tier	
Microscope ($2000)<br>Glucometer ($30)<br>Hemoglobinometer ($300)<br>(Where feasible) Point-of-care ultrasound (cost $6000)	Trained microscopist<br>Trained healthcare worker
Secondary tier (e.g, district-level hospital)	
Automated chemistry analyzer ($75,000-smaller, $110,000-larger)<br>Automated immunoassay analyzer ($56,000 – small; $111,000 larger)<br>Automated hematology analyzer ($110,000)<br>Automated coagulation analyzer ($104,000)<br>Bacterial culture, liquid: ($38,500)<br>Benchtop analyzers:<br>- Blood gas ($10,000)<br>- NAAT ($32,000)<br>- CD4 ($8000)<br>- Microscopes ($2000 each)<br>- Cart-based Ultrasound including multiple transducers, warranty, service contract ($125,000-$150,000)<br>- X-ray ($150,00-$250,000)	Laboratory technician (2 years of training)<br>Laboratory technologist (4 years of training)<br>Laboratory director (desirable: PhD) or Pathologist (desirable: medical degree plus specialty training)<br>Imaging technologist or sonographer (2 years of training)<br>Radiographer (4 years of training)<br>Radiologist (desirable: medical degree plus specialty training)

Note: these include only the equipment costs. Import duties etc add another 25-30%; service contracts not included unless otherwise stated; required building modifications not included
